# Conversion surgery for *BRCA*-mutated pancreatic ductal adenocarcinoma with liver metastasis treated with platinum-based chemotherapy followed by olaparib

**DOI:** 10.1186/s40792-024-01975-x

**Published:** 2024-07-30

**Authors:** Takumi Funo, Daisuke Hashimoto, So Yamaki, Kazuki Matsumura, Hidetaka Miyazaki, Yuki Matsui, Denys Tsybulskyi, Nguyen Thanh Sang, Xu Yaolin, Sohei Satoi

**Affiliations:** 1 Department of Pancreatobiliary Surgery, Kansai Medical University, 2-5-1 Shin-Machi, Hirakata City, Osaka 573-1010 Japan; 2 Division of Surgical Oncology, University of Colorado Anschutz Medical Campus, Aurora, CO USA

**Keywords:** BRCA, Conversion surgery, Liver metastasis, Olaparib, Pancreatic ductal adenocarcinoma

## Abstract

**Background:**

With recent dramatic developments in chemotherapy, attempts to incorporate surgery into the multidisciplinary treatment of unresectable pancreatic ductal adenocarcinoma with metastasis (UR-M PDAC) have emerged. Maintenance therapy with olaparib after chemotherapy including a platinum-based regimen, which inhibits the poly ADP-ribose polymerase (PARP) involved in DNA repair, was approved for UR-M PDAC with positive *BRCA* mutations.

**Case presentation:**

A 47-year-old male patient with a high carbohydrate antigen 19-9 (CA19-9) level was diagnosed with PDAC in the pancreatic tail. Staging laparoscopy revealed occult liver metastasis. Because *BRCA2* mutation was confirmed, triple combination chemotherapy with SOXIRI (S-1/oxaliplatin/irinotecan) was introduced and continued for 16 weeks, followed by 14 weeks of olaparib. After that, CA19-9 was normalized, and no obvious liver metastases of any size could be seen on imaging studies during chemotherapy. Since staging laparoscopy after chemotherapy proved that the liver metastasis had disappeared, laparoscopic distal pancreatectomy was performed, and curative resection was completed. After adjuvant chemotherapy with olaparib for 12 months, the patient is alive 36 months from his initial diagnosis and 27 months postoperatively without recurrence.

**Conclusion:**

We report a case of PDAC with liver metastasis and *BRCA* mutation-positivity who underwent conversion surgery and achieved long-term survival after irinotecan-based chemotherapy followed by maintenance therapy with olaparib.

## Background

Pancreatic ductal adenocarcinoma (PDAC) is a typical refractory malignancy, and at diagnosis, many patients have distant organ metastases, such as liver metastasis and peritoneal dissemination, resulting in a poor prognosis [1, 2]. Current guidelines recommend systemic chemotherapy as the standard treatment for unresectable PDAC with distant organ metastasis (UR-M), such as combination therapy with gemcitabine and nab-paclitaxel or combination therapy with folinic acid, 5-fluorouracil, and irinotecan plus oxaliplatin (FOLFIRINOX) [3–6].

With these recent developments in chemotherapy, some patients have experienced significant shrinkage or disappearance of metastatic lesions, and surgical treatment, so-called “conversion surgery”, has been attempted for such patients [7, 8].

Recently, maintenance therapy with olaparib, which inhibits the poly ADP-ribose polymerase (PARP) involved in DNA repair, was approved for UR-M PDAC with mutations in the *BRCA1/2* gene [9].

This article reports a case in which liver metastases disappeared with S-1/oxaliplatin/irinotecan (SOXIRI) therapy [10] and subsequent olaparib, allowing conversion surgery to be performed. Because olaparib is only effective in *BRCA* mutation-positive patients, cases in which multimodal treatment including olaparib has led to conversion surgery are extremely rare.

## Case presentation

**Fig. 1:**
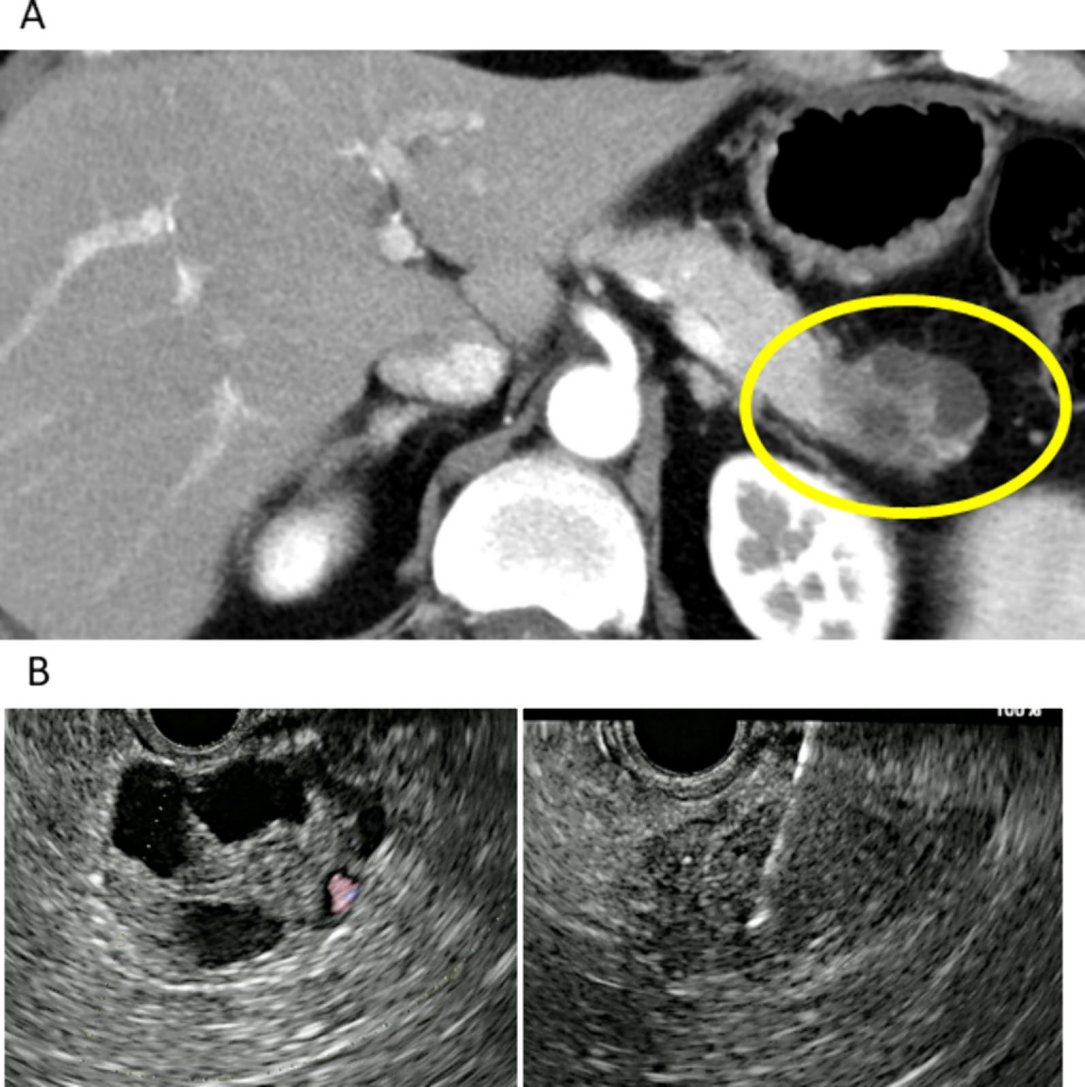
**A** Enhanced computed tomography and **B** endoscopic ultrasonography at the initial diagnosis. **A** The yellow circle shows the primary tumor. **B** The left panel shows a cystic lesion, and the right panel shows endoscopic ultrasonography-guided fine needle aspiration for a solid tumor

A 47-year-old male patient was referred to us because of a high carbohydrate antigen 19-9 (CA19-9) level (50.5 IU/ml). Contrast-enhanced computed tomography scan revealed a 45-mm solid tumor with a cystic lesion in the pancreatic tail with no vascular invasion or distant metastasis (Fig. 1A). Endoscopic ultrasonography (EUS) also showed a solid tumor with a cystic lesion (Fig. 1B). EUS-guided fine needle aspiration was performed, and adenocarcinoma was detected. The patient was diagnosed with resectable (R) PDAC, T3N0M0 stage IIA. The patient underwent *BRCA* mutation examination at the time of the initial visit and was positive for *BRCA2* mutation.

**Fig. 2:**
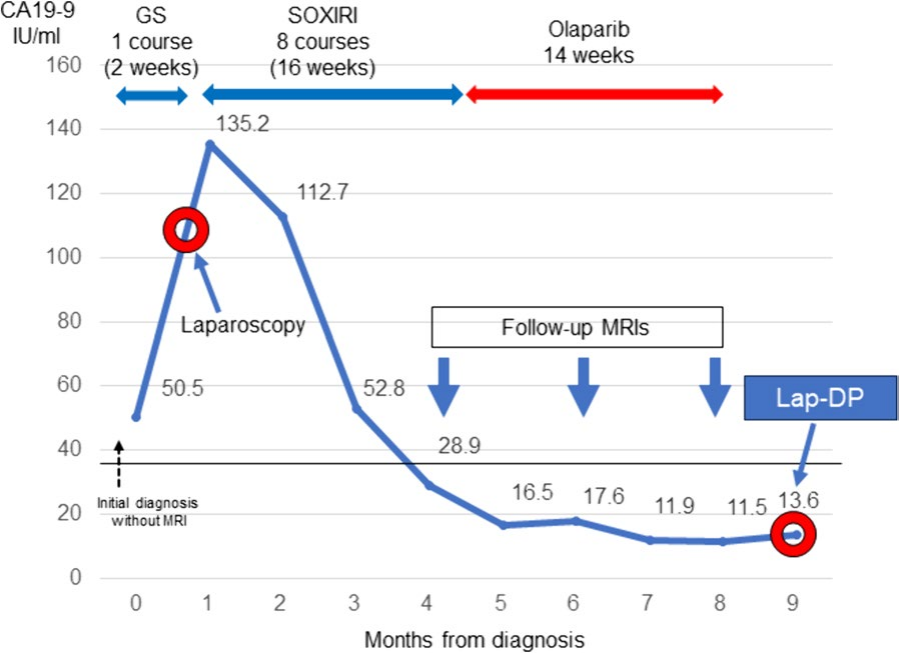
CA19-9 levels during the clinical course of the patient. CA19-9, carbohydrate antigen 19-9; GS, gemcitabine + S-1; Lap-DP, laparoscopic distal pancreatectomy; MRI, magnetic resonance imaging; SOXIRI, S-1/oxaliplatin/irinotecan

**Fig. 3:**
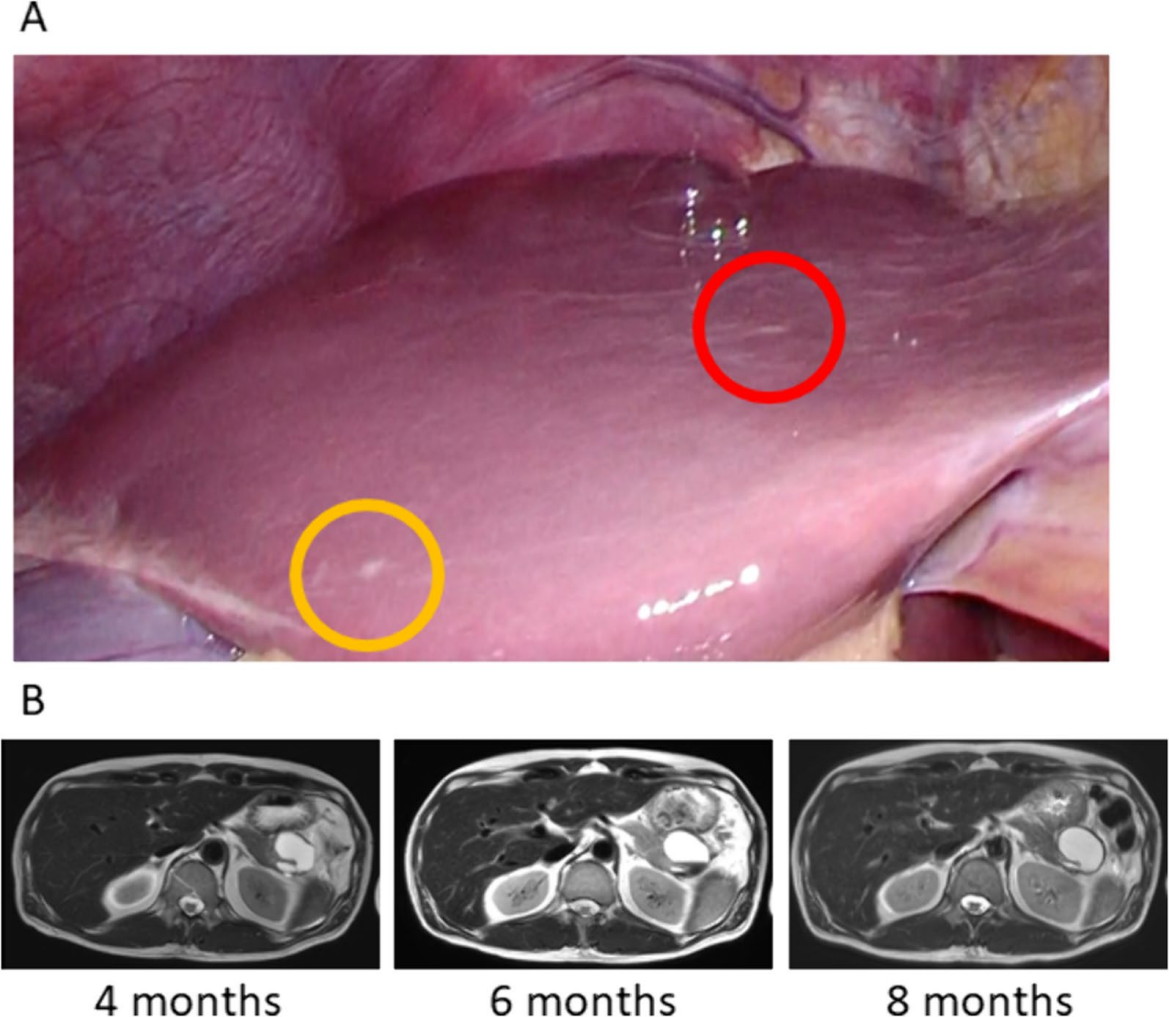
**A** Laparoscopy after 1 course of neoadjuvant gemcitabine + S-1 therapy and **B** gadolinium-ethoxybenzyl-diethylenetriamine pentaacetic acid magnetic resonance imaging during intensive chemotherapy. **A** The circles show small nodules on the liver surface. One (red circle) was diagnosed as a liver metastasis during intraoperative rapid pathological examination. **B** No obvious liver metastases of any size appeared in follow-up imaging. “Months” indicates the time from initial treatment

As neoadjuvant treatment, gemcitabine + S-1 (GS) therapy was introduced, and two courses were scheduled. However, the allergic reaction occurred shortly after the first dose of gemcitabine was started. This patient had not taken S-1, and GS therapy was terminated due to this allergic reaction (skin rash, Common Terminology Criteria for Adverse Events [CTCAE] grade 2) to gemcitabine (Fig. 2) [11]. Neoadjuvant therapy was discontinued without further treatment, and a laparoscopic distal pancreatectomy (DP) was planned. However, laparoscopy revealed two small nodules of the same shape on the liver surface (Fig. 3A). One was intraoperatively diagnosed as a liver metastasis by pathology (Fig. 3A, red circle). Abdominal lavage cytology was negative. Surgery was aborted, and the patient was diagnosed with UR-M PDAC, T2N0M1 stage IV [4], and intense chemotherapy was introduced.

At this point, the patient’s CA19-9 level had risen to 135.2 IU/ml. Because *BRCA2* mutation was confirmed in a prior genetic test, triple combination chemotherapy with SOXIRI was introduced and continued for 16 weeks (8 courses) (Fig. 2). During SOXIRI therapy, thrombocytopenia (CTCAE grade 3) and peripheral neuropathy (CTCAE grade 1) were observed [11]. The CA19-9 level decreased to within normal limits at 4 months after the initial treatment. Following that treatment, olaparib was initiated as a maintenance therapy and continued for 14 weeks (Fig. 2). During olaparib therapy, only diarrhea (CTCAE grade 1) was observed [11]. The CA19-9 level remained within normal limits (Fig. 2), and no obvious liver metastases of any size appeared on gadolinium-ethoxybenzyl-diethylenetriamine pentaacetic acid magnetic resonance imaging (Gd-EOB-MRI) during chemotherapy (Fig. 3B). There was no change in the size of the primary lesion. Therefore, laparoscopic pancreatectomy was planned as a conversion surgery.

**Fig. 4:**
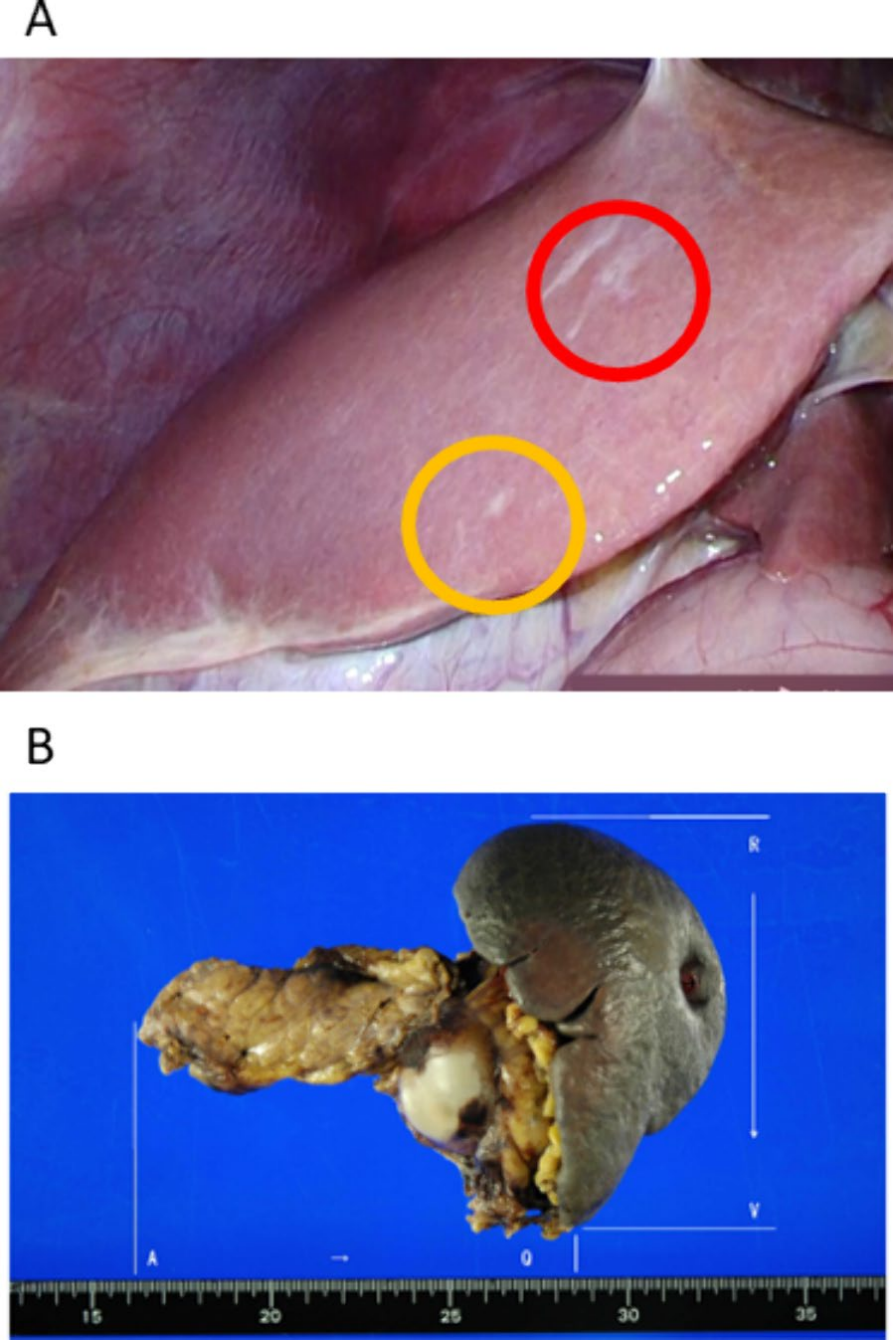
**A** Laparoscopy after intensive chemotherapy including olaparib and **B** a resected specimen after a laparoscopic distal pancreatectomy. **A** The yellow circle shows a small nodule on the on the liver surface that was diagnosed with no malignant findings during intraoperative rapid pathological examination. The red circle was a scar from a biopsy performed during the first laparoscopy. **B** A white cystic lesion is seen in the pancreatic tail

Laparoscopy found two nodules on the liver surface; one was a scar from a biopsy performed during the first laparoscopy (Fig. 4A, red circle). Another nodule was diagnosed intraoperatively with no pathologic malignant findings. (Fig. 4A, yellow circle). Therefore, it was determined that there were no liver metastases, and a laparoscopic DP was performed with radical antegrade modular pancreatosplenectomy method [12] (Fig. 4B). D2 level lymph node dissection was performed, and the pancreatic parenchyma was dissected with an automatic triple-row linear stapler. The operative time was 417 min, and operative bleeding was 362 g. The patient was discharged on postoperative day 9 without any postoperative complications. Pathological diagnosis was well differentiated adenocarcinoma, ypT3N0M0 stage IIA. R0 resection was confirmed, and the distance from the surgical margin was 2000 μm. The College of American Pathologists score was 2, and the Evans grade was III.

One month after surgery, olaparib was started as adjuvant therapy and continued for 12 months. No adverse events were reported during adjuvant therapy. It has now been 36 months since the initial treatment and 27 months since the conversion surgery, and the patient is alive without tumor recurrence.

## Discussion

In this case, a patient who had PDAC with liver metastases at the initial diagnosis underwent conversion surgery after multidisciplinary treatment. To the authors' knowledge, this is the first report of a case in which conversion surgery was performed after olaparib therapy.

In the initial diagnosis and staging of PDAC, contrast-enhanced CT is generally the first step, and Gd-EOB-MRI and positron emission tomography (PET) -CT are performed as needed to scrutinize distant metastases [3]. We do not routinely perform Gd-EOB-MRI or PET–CT in all patients of PDAC. Even if we had, we do not know whether we would have detected the very small liver metastasis in this case, the so-called occult abdominal metastasis (OAM) [1]. After the first surgery in which micro liver metastases were discovered, the patient was followed up with MRI, which is suitable for identification of liver metastases. We have previously shown that PDAC patients with tumor diameter greater than 30 mm, CA19-9 greater than 150 IU/ml, and localization in the pancreatic body and tail are at greater risk of having OAM, and we recommended staging laparoscopy in such cases [13]. Although this patient did not undergo staging laparoscopy in favor of starting chemotherapy early, if it had been performed, it is possible that UR-M would have been diagnosed before the start of neoadjuvant treatment.

In 2020, Golan et al. published the results of a randomized, double-blind, phase 3 trial examining the efficacy of olaparib in patients with *BRCA* mutation-positive metastatic PDAC that had not progressed during first-line platinum-based chemotherapy [9]. The median progression-free survival (PFS), as a primary endpoint, was significantly longer in the olaparib group than in the placebo group (7.4 months vs. 3.8 months, respectively; hazard ratio for disease progression or death, 0.53; 95% confidence interval [CI], 0.35–0.82; *P* = 0.004). The trial also showed that the adverse effect profile of maintenance olaparib was similar to that observed in other tumor types. Health-related quality of life was preserved with olaparib.

Olaparib provides a survival benefit to patients with a germline *BRCA* mutation and metastatic PDAC that did not progress during platinum-based chemotherapy. When the chemotherapy regimen is changed from effective platinum-based therapy to maintenance olaparib with expected prolonged survival and less frequent adverse events, careful follow-up is required to monitor the patient for tumor progression.

The most frequently used platinum-based chemotherapy is FOLFIRINOX. In 2019, we reported the efficacy of SOXIRI therapy, which replaces 5-fluorouracil in FOLFIRINOX with S-1, in a two-center, single-arm, phase II trial for UR-M and locally advanced unresectable (UR-LA) PDAC [10]. The response rate, as a primary end point, was 22.8% (95% CI: 10.4–40.1); median overall survival (OS) was 17.7 months (95% CI: 9.8–22.0), and median PFS was 7.4 months (95% CI: 4.2–8.4). Thus, SOXIRI is considered a promising regimen in patients with unresectable PDAC. The patient described in this case report received SOXIRI therapy as platinum-based chemotherapy.

Surgical resection for liver-only synchronous metastases of PDAC, as in this case, remains controversial. Moreover, there are two major approaches, surgery-first and chemotherapy-first approaches, to the surgical resection of UR-M PDAC [8]. The former involves resection of a small number of distant organ metastases (oligometastasis) with the primary tumor without preoperative chemotherapy or after a short course of preoperative chemotherapy [14]. With the chemotherapy-first approach, after a period of multidisciplinary treatment, the primary tumor is resected in patients in whom distant metastases have disappeared or marked shrinkage is noted on imaging studies, so-called “conversion surgery” [7]. The OS of patients treated with the surgery-first approach was reported to range from 5.9 to 21.9 months [15–26]. On the other hand, the median OS was 34.1 to 56.0 months from the initiation of chemotherapy and 24.4 to 46.0 months after conversion surgery [18, 27, 28]. Thus, OS is apparently better with conversion surgery (chemotherapy-first approach) than with synchronous resection of the primary tumor and liver metastasis (surgery-first approach). Consequently, this patient was treated with the chemotherapy-first approach, and conversion surgery was performed successfully.

Our criteria for conversion surgery for UR-M PDAC were: anatomical resectability (absence of liver metastases in this patient), biological response to chemotherapy (CA19-9 in this patient), and good general condition of the patient. In addition, the appropriate timing for conversion surgery for initially UR-M PDAC has been still unclear. We do not currently define the duration of preoperative chemotherapy as an indication for conversion surgery. However, considering the results of previous studies, the duration of induction chemotherapy for decision-making regarding conversion surgery in patients with UR-M has been reported to be longer than at least 8–9 months for confirming disease progression (depending on tumor marker levels), resulting in favorable OS in patients with UR-M PDAC [29–33].

This patient had two liver nodules of the same nature, one of which was diagnosed as a liver metastasis at the first surgery (Fig. 3A, red circle). The other was diagnosed as not being a liver metastasis at the second surgery (Figs. 3A and 4A, yellow circle). It is possible that the other nodule (Figs. 3A and 4A, yellow circle) was not a liver metastasis from the beginning, but what is important is that there was no liver metastasis at the second surgery after 30 weeks of chemotherapy with SOXIRI and olaparib.

The significance of adjuvant chemotherapy after conversion surgery remains unclear. Whereas several previous studies of conversion surgery showed better patient survival with adjuvant therapy than without it [17, 29, 30], the appropriate regimen of adjuvant therapy after surgery for UR-M PDAC remains unknown [8]. There is no clear evidence to verify this. The standard regimen for postoperative adjuvant therapy for PDAC in Japan is S-1 for six months, but the clinical trials in which this was established did not include conversion surgery for UR-M PDAC [34]. It is unclear whether it is better to use the guideline-recommended regimen as adjuvant therapy or to again use the regimen that was effective for distant metastases in the preoperative treatment as adjuvant therapy. The patient in this report took olaparib for one year and had no recurrence. No evidence exists for the use of olaparib as adjuvant therapy, and it should be carefully studied in the future.

## Conclusion

We report a case of PDAC with liver metastasis revealed by laparoscopy and BRCA mutation-positivity who underwent conversion surgery and achieved long-term survival after multimodal treatment with SOXIRI, a platinum-based chemotherapy, followed by olaparib maintenance therapy.

## Data Availability

Data sharing does not apply to this article because no datasets were generated or analyzed.

## Abbreviations

CA19-9: Carbohydrate antigen 19-9
CI: Confidence interval
CTCAE: Common Terminology Criteria for Adverse Events
DP: Distal pancreatectomy
EUS: Endoscopic ultrasonography
FOLFIRINOX: Folinic acid, 5-fluorouracil, and irinotecan plus oxaliplatin
Gd-EOB-MRI: Gadolinium-ethoxybenzyl-diethylenetriamine pentaacetic acid magnetic resonance imaging
GS: Gemcitabine + S-1
PARP: Poly ADP-ribose polymerase
PDAC: Pancreatic ductal adenocarcinoma with metastasis
PFS: Progression-free survival
OS: Overall survival
SOXIRI: S-1/oxaliplatin/irinotecan
UR-LA: Locally advanced unresectable
UR-M: Unresectable with metastasis
